# Items

**Published:** 1878-08

**Authors:** 


### Items.

THE LAST ISSUE of the *Cincinnati Lancet and Observer* announces the consolidation of that journal and *The Clinic.* The combination will be known as *The Cincinnati Lancet and Clinic* and will be under the control of the efficient editors of the two journals: Dr. J. G. Hyndman, of the *Clinic*, and Dr. J. C. Culbertson and Dr. T. M. Stevens, of the *Lancet and Observer.*

TO SUBSCRIBERS.—We frequently receive letters announcing that a certain number of the JOURNAL has not been received and, on referring to our books, we find that the subscriptions of the writers of these letters have not been paid. Of course mistakes will occur and some of our subscribers who have paid, may occasionally be overlooked; but if every one would look at his receipt before sending for missing numbers, and to such intimation add the amount of his subscription, it would greatly facilitate the business of the publishing office.

THE TRADITIONS OF THE PAST are fast dissolving in the retorts of the modern investigator. And now come Foersch, Horsfield and others who show that the upas tree (antiaris toxicaria) is far from possessing the deadly qualities which it has so long possessed in English metaphor. The pestiferous influences which it was once supposed to exert, really originated in the sulphurous and other vapors emitted from the volcanic regions in which the upas tree is discovered. It is true that the branches, flowers and inner bark of the young trees may excite itching of the skin when handled, and the dried juice mixed with other ingredients, forms the poison in which the natives of the Indian Archipelago dip their arrows. Travellers have, however, considerably exaggerated the effects produced by wounds from these medicated darts. It is quite certain that the statements are gross exaggerations which describe the plant as growing in a desert tract with no other plant near it for ten or twelve miles, criminals offered a pardon if they would collect its poison, the ground in its vicinity strewn with the bones of those who ventured too near, and the destruction of birds, fish, and vermin within a large area over which the effluvium of the upas tree was supposed to extend.

THE *Pacific Medical and Surgical Journal* says: "Chicago must be a paradise for ignorant druggists. The CHICAGO MEDICAL JOURNAL tells of one who pursued the business of a druggist in order to sell liquors, and who dispensed strychnine instead of arsenic, nearly killing the person who took it. * * * What is the Chicago College of Pharmacy about?"

The Chicago College of Pharmacy, we believe, is about its own business, and is nowise responsible for ignorant druggists in *Dover*, Ill. This is the name of the place given by us in connection with the above story. It was not Chicago, for here such sham druggists could not live twenty-four hours.

KARL ROKITANZKY, Professor of Pathological Anatomy at the University of Vienna, died at Vienna on the 23d of July. Rokitanzky was born in Kœniggrætz, Bohemia, February 19, 1804. Having received a liberal education at his native place, he studied medicine in Prague and Vienna. At the latter place he graduated in 1828, established himself as "Privatdocent" in 1832, was made *professor extraordinarius* in 1834, and elected *professor ordinarius* of pathological anatomy in 1844. His professorship gave him the full control over the dead-house of the Vienna General Hospital, where vast material offered a rare opportunity for scientific researches. How well he has understood to utilize this material is shown by his numerous contributions to medical journals, but chiefly by that standard work on morbid anatomy which carried his fame all over the world. This "Handbook of Pathological Anatomy" was published in Vienna, between 1842 and 1846, in 3 volumes, translated into English by the Sydenham Society, 1845 to 1850. Rokitanzky is the founder of the "New Vienna Medical School," which justly attracted numerous students from all parts of the globe, for seldom one school can boast of such a galaxy of names as Rokitanzky, Skoda, Oppolzer, Hebra, Schuh, Dietrich, Hamernjk and others.

